# Runting and Stunting Syndrome Is Associated With Mitochondrial Dysfunction in Sex-Linked Dwarf Chicken

**DOI:** 10.3389/fgene.2019.01337

**Published:** 2020-01-17

**Authors:** Hongmei Li, Bowen Hu, Qingbin Luo, Shuang Hu, Yabiao Luo, Bojing Zhao, Yanmin Gan, Ying Li, Meiqing Shi, Qinghua Nie, Dexiang Zhang, Xiquan Zhang

**Affiliations:** ^1^ Department of Animal Genetics, Breeding and Reproduction, College of Animal Science, South China Agricultural University, Guangzhou, China; ^2^ Guangdong Provincial Key Lab of Agro-Animal Genomics and Molecular Breeding and Key Lab of Chicken Genetics, Breeding and Reproduction, Ministry of Agriculture, College of Animal Science, South China Agricultural University, Guangzhou, China; ^3^ Institute of Animal Science, Guangdong Academy of Agricultural Sciences, Guangzhou, China; ^4^ Division of Immunology, Virginia-Maryland Regional College of Veterinary Medicine, University of Maryland, College Park, College Park, MD, United States

**Keywords:** runting and stunting syndrome, chicken, mitochondrial dysfunction, oxidative phosphorylation, ATP synthesis, vacuoles

## Abstract

Runting and stunting syndrome (RSS) in chicken are commonly known as “frozen chicken.” The disease is characterized by lower body weight and slow growth and the incidence rate is widely 5%–20% in sex-linked dwarf (SLD) chickens. However, the etiology of RSS in chickens has plagued researchers for several decades. In this study, histopathology studies demonstrated that the hepatocytes of the RSS chickens contain many mitochondria with damaged and outer and inner membrane along with vacuolar hydropic degeneration. No mtDNA mutation was detected, but our microarray data showed that RSS chickens exhibited abnormal expression of genes, many of which are involved in oxidative phosphorylation (OXPHOS) and fatty acid metabolism. In particular, nuclear gene *IGF2BP3* was upregulated in RSS chickens' liver cells. The abnormal expression of these genes is likely to impair the OXPHOS, resulting in reduced ATP synthesis in the hepatocytes of the RSS chickens, which may in turn leads to poor weight gain and retarded growth or stunting of chicks. Our findings suggest that mitochondria dysfunction rather than chronic inflammation is responsible for the reduced growth and RSS in SLD chickens. Mutations in *GHR* have been shown to compromise mitochondrial function in SLD chickens. Since the mitochondrial damage in the RSS chicken is more severe, we suggest that extra genes are likely to be affected to exacerbate the phenotype.

## Introduction

Runting and stunting syndrome (RSS) is a condition in which a number of the birds in a flock are considerably smaller owing to delayed growth (Zavala, 2006), and RSS chickens are easily observed in sex-linked dwarf (SLD) chickens. It is known that the SLD chickens are caused by the mutation of growth hormone receptor (*GHR*) gene and characterized by shorter shanks, and lower body weight (Ouyang et al., 2012). But the RSS affected SLD chickens are smaller and lower body weight than SLD chickens. RSS has been reported in poultry flocks around the world, including in the Netherlands (Kouwenhoven et al., 1978) and the UK (Bracewell and Wyeth, 1981). RSS causes economic hardship in the poultry industry through reduced uniformity, increased mortality, decreased body weight, poor feed conversion rate, and numerous secondary diseases. The first clinical signs may be observed as early as three days of age, although they are most commonly observed in 6- to 12-day-old chicks and may occur until 3 weeks of age. The chicks are frequently pale and may exhibit distention of the abdomen. In addition to smaller size and reduced body weight, other clinical signs were also observed in RSS chickens include poor feather development, listlessness, and diarrhea. The comb develops slowly, which is associated with impaired hearing and vision.

Why are RSS chickens easily observed in SLD chickens? Our study demonstrated the occurrence of mitochondrial abnormalities in the 7-week-old chicken's livers of RSS chickens and SLD chickens. RSS chicken's mitochondria dysfunction is more seriousness than SLD chickens, and we observed the abnormal nuclear genes related to mitochondrial function in 7-week-old RSS chickens and normal SLD chickens by expression patterns (**Table 2**), and found these genes involved in oxidative phosphorylation (OXPHOS) and other metabolic pathways.

The chicken mitochondrial genome is a 16.775-kb circle of double-stranded DNA (Desjardins and Morais, 1990; Zavala, 2006) that encodes only 13 proteins, 2 rRNAs, and 22 tRNAs, which is very similar to the human mitochondrial genome (Desjardins and Morais, 1990). It has been reported that mutation or deletion of mitochondrial DNA (mtDNA) can induce mitochondrial disorders in humans (Zeviani et al., 1989; Moraes et al., 1991). As over 99% of mitochondrial proteins are encoded by the nuclear genome, mitochondrial disorders can also be caused by mutations or/and abnormal expression of nuclear-encoded mitochondrial proteins (Bourgeron et al., 1995). In this study, we detected no mtDNA mutations in RSS chickens and SLD chickens.

Very little is currently known about mitochondrial diseases of chickens, and the etiology of RSS in chickens has plagued researchers for several decades. OXPHOS is the metabolic pathway in which cells use enzymes to oxidize nutrients, thereby releasing energy that is used to reform adenosine triphosphate (ATP). This pathway is a highly efficient way of generating energy, compared to alternative fermentation processes such as anaerobic glycolysis. OXPHOS also produces reactive oxygen species (ROS) which lead to the propagation of free radicals, damaging cells, and contributing to disease and possibly aging. The enzymes responsible for this metabolic pathway are also the targets of many drugs that inhibit their activities.

In this study, we demonstrated that mitochondrial membranes are destroyed in RSS chicken hepatocytes using transmission electron microscopy (TEM). Activity assays revealed that a series of complexes proteins (complex I, complex II, complex III, and complex IV) showed decreased enzyme activities in RSS chickens and SLD chickens, suggesting that the OXPHOS function was compromised.

## Materials and Methods

### Ethics Standards

All of the animal experiments performed in this study were approved by South China Agriculture University's Institutional Animal Care and Use Committee (approval number SCAU#0017), according to the regulations established by this committee and international standards for animal welfare.

### Animals

The chickens were purchased from XinXing Poultry Breeding Company near Guangzhou, China. Three groups of chickens were used: RSS affected SLD chickens (RSS chickens) along with normal SLD chicken (SLD chickens) in 301 strain, and this strain is characterized by point mutations in exon 5 of the *GHR* gene as previously reported (Ouyang et al., 2012); normal chickens in 202 strain, which have a wild-type *GHR* gene. All of the chicks were provided with standard poultry feed and fresh water and reared at an optimum temperature (27°C–37°C). After 7 weeks, six RSS chickens (three males and three females) and 6 SLD chickens (3 males and 3 females) from the 301 strain group, along with six normal chickens (three males and three females) from the 202 strain group, were collected. All of the experimental chicks were monitored periodically for signs of RSS, including body weight, abnormal feathering, uneven growth rate, poor performance, beak and leg color, lameness, and reluctance to move.

### Liver Histology

Liver tissues were removed from each experimental chicken and fixed in formalin and 2.5% glutaraldehyde, respectively. The samples were stained using hematoxylin and eosin (H&E) and Oil Red O, the slices were observed by optical microscopy, and photographed. We also used TEM (Hitachi HT-7700, Japan) to study the mitochondrial structure.

### Transmission Electron Microscopy

Liver tissues were fixed in 2.5% glutaraldehyde for 4 h at 4°C and then cultured as previously described (Luo et al., 2016). Transmission electron microscope (Hitachi HT7700, Japan) was used to examine and photograph mitochondria, and five randomly selected areas were photographed at × 2500 magnification and counted as previously reported (Hu et al., 2019).

### Quantitative Real-Time PCR

To verify the data from microarray analysis (**Table 2**), quantitative real-time PCR (qRT-PCR) analysis was conducted to determine the relative expression levels of genes involved in the OXPHOS pathway in the livers of RSS chickens, SLD chickens, and normal chickens. Total RNA was extracted from the tissue samples using the RNAiso reagent (Takara, Japan) according to the manufacturer's instructions. The RNA integrity and concentration were determined using 1.5% agarose gel electrophoresis and a Nanodrop 2000c spectrophotometer (Thermo, USA), respectively. cDNA was carried out using a PrimeScript RT Reagent Kit (Takara) for RT-qPCR. The MonAmp™ ChemoHS qPCR Mix (Monad, China) was used for qRT-PCR in a Bio-Rad CFX96 Real-Time Detection instrument (Bio-Rad, USA) according to the manufacturer's protocol. Relative gene expression was measured by qRT-PCR twice for each reaction and nuclear gene β-actin was used as a control. The primers are listed in **Table 1**.

**Table 1:** Primers for qPCR analysis of nuclear gene expression and mtDNA content.

Gene	Primer sequence (5′ to 3′)	Annealing temperature (°C)
*F-NDUFB1*	TGTACAAGAGAGAGCTGAAGCC	57.5
*R-NDUFB1*	AGCAGTGACAAGTTGTAGGTGT	
*F-NDUFB2*	GCTGGGCCACTTCCCGTA	64
*R-NDUFB2*	AGTTCAAAACCCGCCCCTAC	
*F-NDUFB8*	CGATGCACTGGGACTTTGAC	65
*R-NDUFB8*	TTGTAATGCGTCACAACCGG	
*F-NDUFB9*	GGCAGAAAGAGGTGAAGCAG	65
*R-NDUFB9*	CATACGTTCACTGGCACACC	
*F-NDUFB10*	CCAGCAAGCCAAGAACAAGT	64.3
*R-NDUFB10*	TTGGTAACCTGTGCAAGCT	
*F-NDUFB5*	TGAGACAGAGGGGAGATGGA	64.3
*R-NDUFB5*	CTCCACAGACAAGCCACAAT	
*F-NDUFB6*	GGTGTTCAACGCTTACCAGA	64.3
*R-NDUFB6*	CCGTCTCTAAAATTCTGTCCCC	
*F-IGF2BP3*	CAAGCTCTACATCGGCAACC	65
*R-IGF2BP3*	GGGACCGAATGCTCAACTTC	
*F-NDUFA8*	ACAAGGAGTTCATGCTGTGC	57.5
*R-NDUFA8*	CACCCAGCCCAACTTCTCTA	
*F-NDUFA9*	ATCCACTTCCACGTCCTCTC	65
*R-NDUFA9*	CTTCGCTGGTTTTGCTTCCT	
*F-SDHA*	GGCACTGCTATGGTTACACG	65
*R-SDHA*	CCCCTTCCTTCCCGTATCTC	
*F-NDUFS4*	GGACTGGACTTCTCTGTGCT	57
*R-NDUFS4*	TGGCAGCAGGGAATACAGAA	

### Mitochondrial Protein Concentration

The mitochondrial protein concentration was measured using a BCA protein assay kit (Beyotime, China) carried out in a Fluorescence/Multi-Detection Microplate Reader (BioTek, USA) according to the manufacturer's instructions.

### Enzyme Activities of Mitochondrial Respiratory Complexes

Liver tissues were dissected, frozen in liquid nitrogen, and then stored at −80°C (Thorburn et al., 2004). Enzyme activities of mitochondrial respiratory complexes were measured using commercial assay kits (Solarbio, China) according to the manufacturer's instructions carried out in a Fluorescence/Multi-Detection Microplate Reader (BioTek, USA) according to the manufacturer's protocol. The enzyme activity of complex I was determined by the change in absorbance of NADH measured at 340 nm. The enzyme activity of complex II was determined by the change in absorbance of 2, 6-dichlorophenolindophenol measured at 600 nm. The enzyme activities of complex III and complex IV were determined by the change in absorbance of reduced cytochrome c measured at 550 nm.

### Mitochondrial Respiratory Control Ratio

The mitochondrial respiratory control ratio (RCR) was determined using an RCR assay kit (Genmed Scientifics Inc., USA) according to the manufacturer's instructions as previously described (Su et al., 2016). Oxygen consumption was measured using a Clarke-type oxygen electrode (Hansatech Oxytherm, UK). RCR was represented as the ratio of State III to State IV respiration rate. Data were normalized to the control group and expressed as percentage of control levels.

### ATP Concentration

The ATP level was measured using an ATP assay kit (Beyotime, China) according to the manufacturer's instructions. A Fluorescence/Multi-Detection Microplate Reader (BioTek, USA) was used to determine the ATP level in gastrocnemius muscle and cells. Data were normalized to the control group and expressed as percentage of control levels.

### Mitochondrial Membrane Potential

The mitochondrial membrane potential (ΔΨm) was measured using a JC-1 kit (Beyotime, China) according to the manufacturer's instructions. The fluorescence was determined using a Fluorescence/Multi-Detection Microplate Reader (BioTek, USA). Rotenone was used as standard inhibitor of ΔΨm. The ΔΨm of mitochondria were represented as the ratio of JC-1 aggregated and JC-1 monomeric, and data were normalized to the control group and expressed as percentage of control levels.

### ROS Production

The production of ROS was measured by the change in absorbance of formazan measured at 560 nm as previously described (Kim et al., 2003). Absorbance was determined using a Fluorescence/Multi-Detection Microplate Reader (BioTek, USA).

### Statistical Analysis

All the experiments were performed at least three times. The data were presented as means ± standard error of the mean (S.E.M.), and the statistical analyses were performed using Student's *t*-test and the significance was represented by P-values. P value < 0.05 was considered to be statistically significant.

## Results

### RSS Chickens and SLD Chickens At the Same Age Exhibit Different Growth Stages

**Figure 1:**
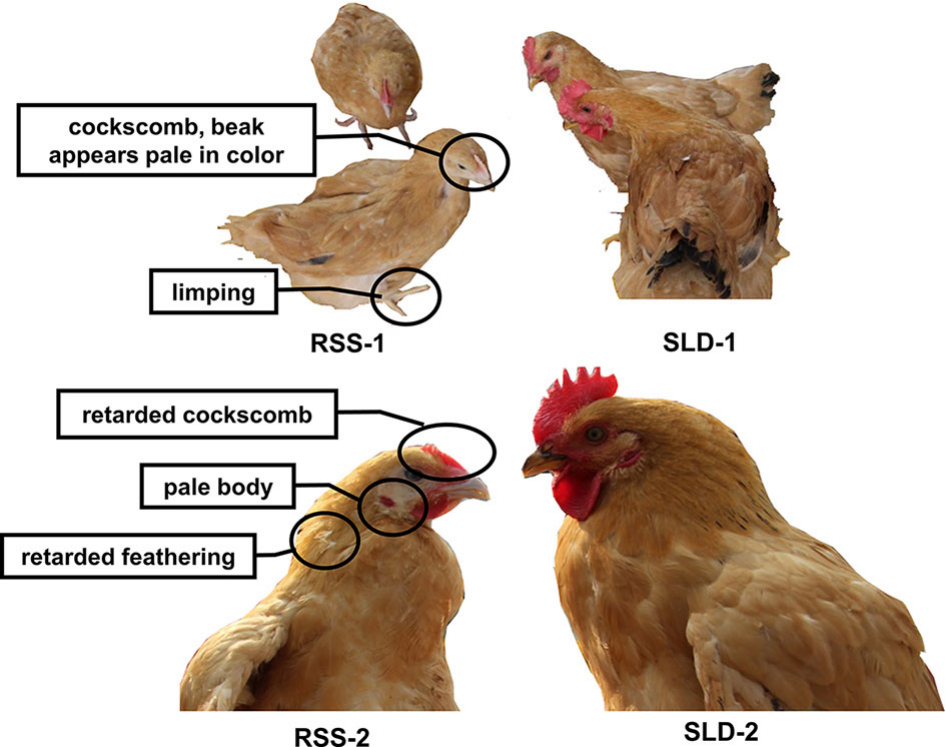
Photographs of runting and stunting syndrome (RSS) chickens and sex-linked dwarf (SLD) chickens at the same age but different growth stages. The RSS chickens are smaller and possess villi faded slowly compared with the SLD chickens, regardless of sex.

As shown in **Figure 1**, RSS chickens were smaller with villi faded slowly compared with SLD chickens. The cockscomb, legs, and beaks of the RSS chickens appeared pale in color, indicating a decline in blood production. Some of the chickens were found to have rickets, broken legs, or fractured toes. In addition, all RSS chickens were ALV-J and REV virus negative showed in **Figure S1**.

### Abnormally Expressed Genes in the Livers of RSS Chickens Compared With SLD Chickens Are Mainly in the OXPHOS Pathway, Fatty-Acid Metabolism Pathway, and Other Metabolic Pathways

We next examined gene expression differences in the livers using expression profile chip analysis. Gene Set Enrichment Analysis results revealed that the RSS chickens exhibited abnormal expression of numerous genes involved in metabolic pathways (**Table 2**). We identified that many of the abnormally expressed genes were involved in OXPHOS pathway. These genes are associated with metabolic function in chickens. The results indicate a defective energy metabolism in RSS chickens compared with SLD chickens.

**Table 2:** Abnormally expressed genes in the livers of runting and stunting syndrome (RSS) chickens [relative to sex-linked dwarf (SLD) chickens].

Pathway	Gene
Oxidative phosphorylation	*NDUFB5, NDUFB6, NDUFB8, NDUFB9, ATP6V1G1, NDUFB1, NDUFB2, ATP6V0C, NDUFS4, ATP5I, TCIRG1, NDUFB10, NDUFA8, NDUFA9, NDUFA7, ATP5F1, ATP6V1D, LOC770190, PPA1, COX6C, SDHA, ATP6V1E1, COX6A1, ATP6V0A2, LOC770879*
Fatty-acid metabolism	*ALDH7A1, CPT2, ADH5, ACADL, ACAT2, ALDH3A2, ACSL5*
Metabolism of xenobiotics by cytochrome P450	*CYP3A37, ADH5, EPHX1, LOC421447, LOC396380, UGT1A1, CYP3A80*

Complex I is the first complex in the electron transport chain (Hirst, 2005). The results revealed that the RSS chickens exhibited altered expression of the complex I genes. The change of the enzyme complex function directly or indirectly reflects the change of mitochondrial function. Furthermore, the *IGF2BP3* and *SDHA* genes were abnormally expressed in RSS chicken livers.

### H&E Staining and Oil Red O Staining of RSS Chickens, SLD Chickens and Normal Chickens

**Figure 2:**
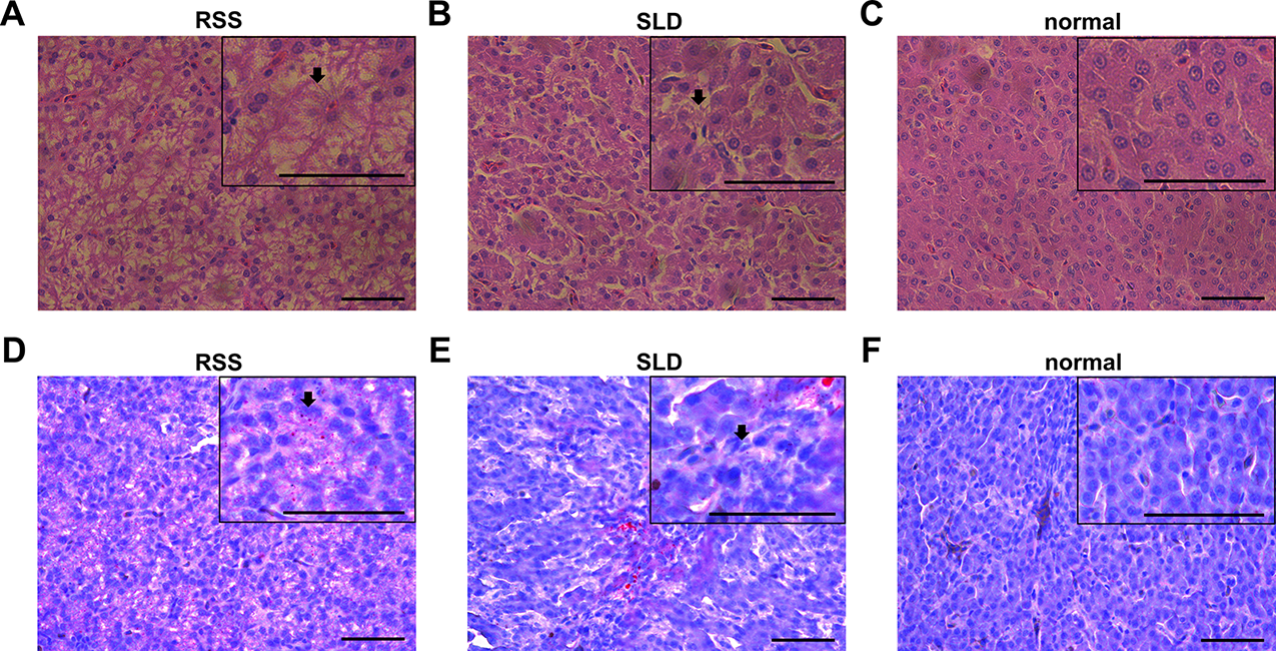
Liver tissues stained with H&E and Oil Red O. **(A)** Liver section stained with H&E from runting and stunting syndrome (RSS) chicken. Bar, 50 μm. As indicated by the arrow, the hepatocytes possess a sparse cytoplasm and contain a large number of vacuoles. The hepatocytes are not only abnormal in terms of morphology and structure but also present in reduced number. **(B)** Liver section stained with H&E from sex-linked dwarf (SLD) chicken. Bar, 50 μm. Compared with the hepatocytes from the RSS chicken, the hepatocytes of the normal SLD chicken contain fewer vacuoles and less sparse cytoplasm. However, compared with the hepatocytes from the normal chicken, the hepatocytes of the SLD chicken possess a sparse cytoplasm and contain a larger number of vacuoles. **(C)** Liver section stained with H&E from normal chicken. Bar, 50 μm. The hepatocytes possess a normal shape and structure, and no vacuoles and sparse cytoplasm are observed. **(D, E)** Liver sections stained with Oil red O from RSS and SLD chickens, respectively, showing vacuolar hydropic degeneration, which leads to an incomplete structure of hepatocytes. Bar, 50 μm. These phenomena were more severe observed in the RSS chickens. **(F)** Liver section stained with Oil red O from normal chicken. Bar, 50 μm. No abnormal vacuoles in the hepatocytes is evident.

Several viruses, bacteria, and other pathogens are believed to be responsible for RSS in broiler chickens. Bacteria are frequently isolated from RSS birds, which include *Escherichia coli*, *Proteus mirabilis*, *Enterococcus faecium*, *Staphylococcus cohnii*, *Clostridium perfringens*, *Bacteroides fragilis*, and *Bacillus licheniformis*. They are commonly found in the intestinal tract and may cause secondary infections that aggravate the initial lesions (Rebel et al., 2006). Some researchers have demonstrated that astrovirus may cause RSS in broilers (Kang et al., 2018). In this study, vacuolation of the hepatocytes was observed in both RSS and SLD chickens (**Figures 2A**–**F**). Hydrophic degeneration in the hepatocytes was also seen in the RSS chickens, which may be responsible for the liver cell vacuoles (**Figures 2A**, **D**). The hydrophic degeneration in the hepatocytes of RSS chickens may disrupt the structure and inhibited the function of mitochondria. This may further result in the destruction of the energy supply functions of the mitochondria.

### Mitochondrial Volume and Mitochondrial Structure of RSS Chickens, SLD Chickens, and Normal Chickens

**Figure 3:**
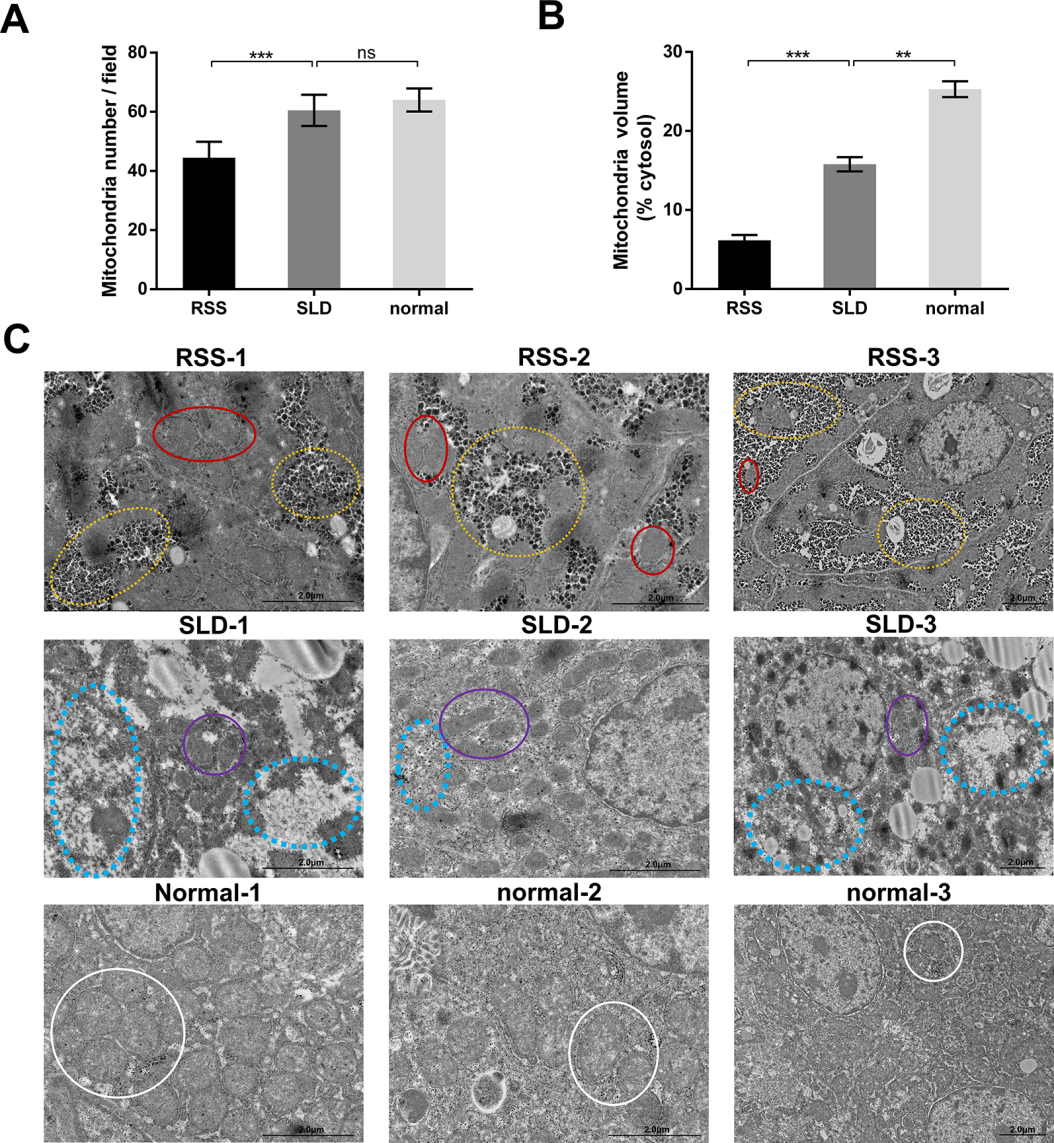
Total and average volumes of mitochondria and transmission electron microscopy (TEM) images. Total volume **(A)** and average volume of mitochondria **(B)** in runting and stunting syndrome (RSS) chickens, sex-linked dwarf (SLD) chickens, and normal chickens. To examine the mitochondrial morphology, five randomly selected areas were imaged at 25,000× magnification and analyzed. **(C)** RSS 1–3: TEM images of liver sections from 7-week-old RSS chickens. In this group, vacuoles in the hepatocytes had destroyed the mitochondria and other organelles, as indicated by the yellow circles. This not only caused a severe decrease in the number of mitochondria, leading to the destruction of the interactions between mitochondria and other organelles, but also seriously affected the structure and function of the mitochondria. As indicated by the red circles, the mitochondria were small in size and their inner and outer membranes had been destroyed or even disappeared. The mitochondrial cristae had also disappeared. SLD 1–3: TEM images of liver sections from 7-week-old SLD chickens. In this group, the mitochondria in the hepatocytes are surrounded by a large number of vacuoles, and the number of mitochondria is also reduced. The mitochondria are small in size and their structure is incomplete, although the degree of damage is less than that in the RSS chickens. Vacuoles and parts of destroyed mitochondria are indicated by blue and purple circles, respectively. Normal 1–3: TEM images of liver sections from 7-week-old normal chickens. In this group, normal mitochondria (indicated by white circles) and other organelles are observed. They are not only complete in structure but also present in large numbers. Data are represented as the mean ± SEM; **p < 0.01; ***p < 0.001; ns, no significan difference.

We next analyzed the structure of the mitochondria using TEM. The results revealed that the total volume and average volume of mitochondria which were reduced in RSS chickens compared with SLD chickens and normal chickens (**Figures 3A**, **B**). Furthermore, excessive amounts of vacuoles were present in the hepatocytes of RSS chickens. This may induce a decrease in the number of mitochondria. The inner and outer mitochondrial membranes exhibited different degrees of damage, and even disappeared, and the endoplasmic reticulum (ER) structure was also damaged (**Figure 3C**).

### Expression of Genes Involved in the OXPHOS Assessed by qRT-PCR

**Figure 4:**
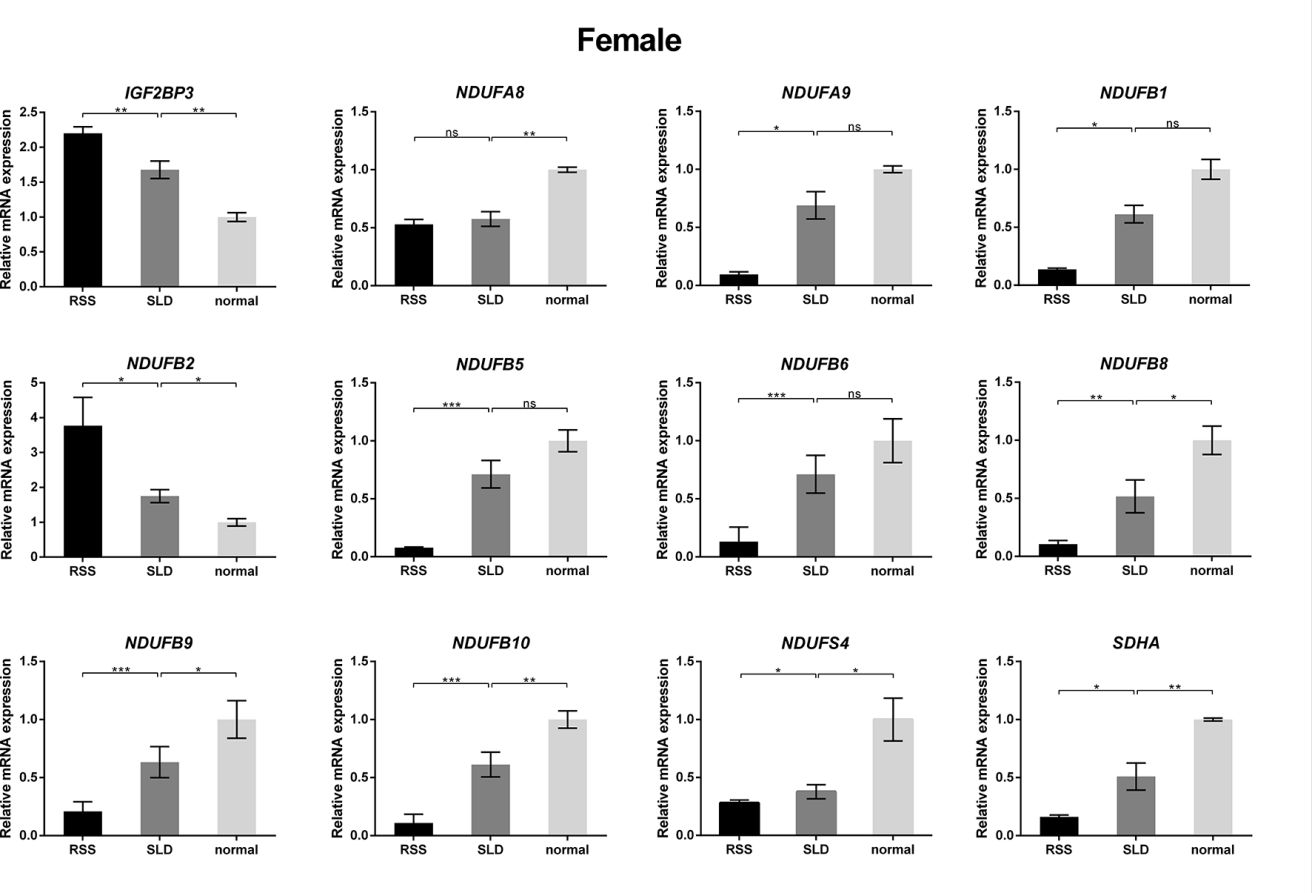
Relative expression of *IGF2BP3* and other genes involved in oxidative phosphorylation (OXPHOS) in the livers of female chickens. Comparison of the female runting and stunting syndrome (RSS) chickens with sex-linked dwarf (SLD) chickens and SLD chickens with normal chickens revealed that the *IGF2BP3* and *NDUFB2* genes were upregulated and the *NDUFA8*, *NDUFA9*, *NDUFB1*, *NDUFB5*, *NDUFB6*, *NDUFB8*, *NDUFB9*, *NDUFB10*, *NDUFS4*, and *SDHA* genes were downregulated. Data are represented as the mean ± SEM; *p < 0.01; **p < 0.01; ***p < 0.001; ns, no significant difference.

**Figure 5:**
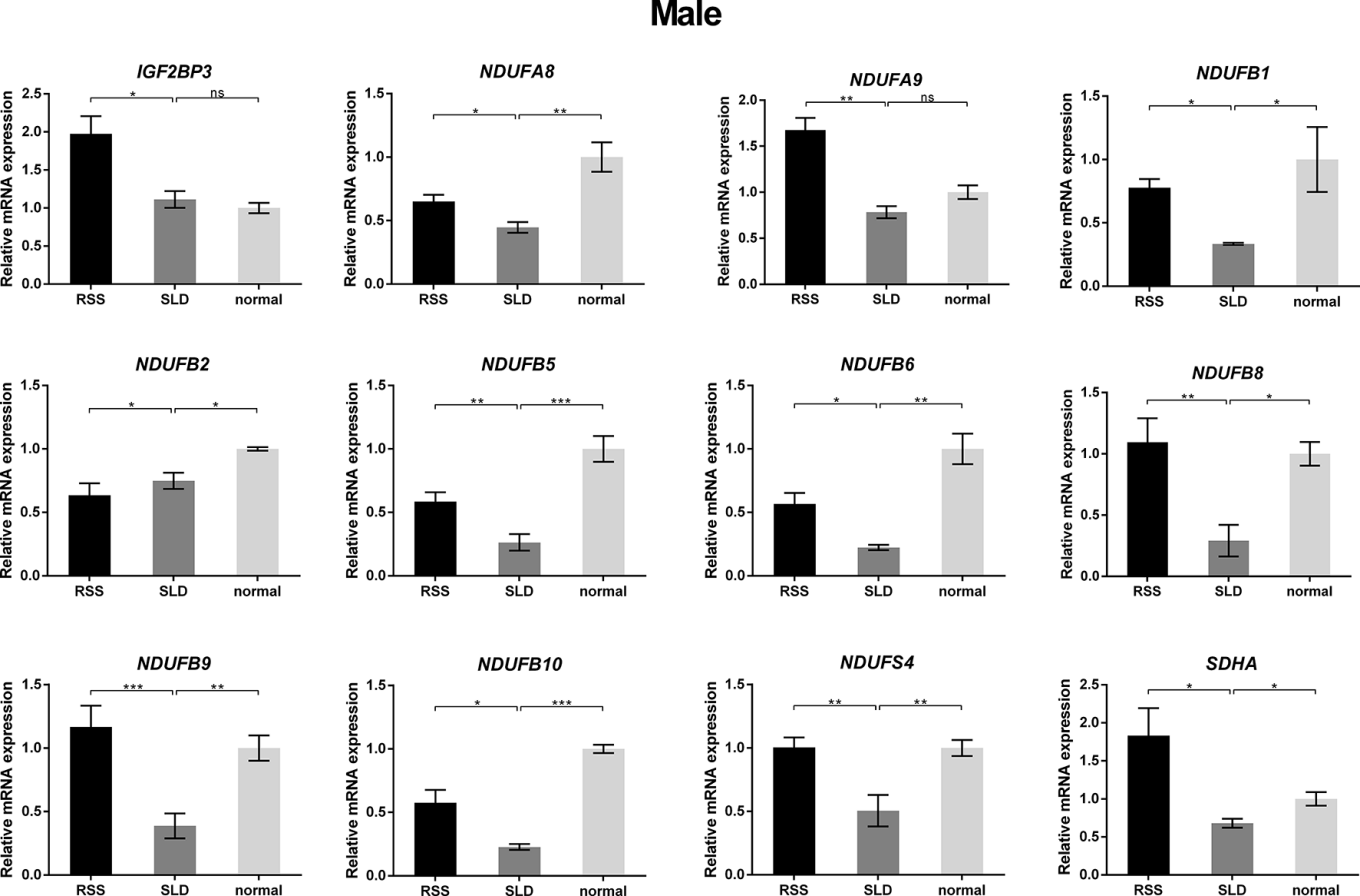
Relative expression of *IGF2BP3* and other genes involved in oxidative phosphorylation (OXPHOS) in the livers of male chickens. Comparison of the male RSS chickens with SLD chickens the *IGF2BP3*, *NDUFA8*, *NDUFA9*, *NDUFB1*, *NDUFB5*, *NDUFB6*, *NDUFB8*, *NDUFB9*, *NDUFB10*, *NDUFS4*, and *SDHA* genes were upregulated and the *NDUFB2* genes were downregulated. Comparison of the male SLD chickens with normal chickens the *IGF2BP3* were upregulated and the *NDUFA8*, *NDUFA9*, *NDUFB1*, *NDUFB2*, *NDUFB5*, *NDUFB6*, *NDUFB8*, *NDUFB9*, *NDUFB10*, *NDUFS4*, and *SDHA* genes were downregulated. Data are represented as the mean ± SEM; *p < 0.01; **p < 0.01; ***p < 0.001.

To verify the consequences of expression profile chip analysis, the relative expression levels of genes involved in mitochondrial OXPHOS were determined in RSS chickens, SLD chickens, and normal chickens. These three groups of chickens were evaluated at 7 weeks of age to verify the microarray data. As shown in **Figure 4**, comparison of the female RSS chickens with SLD chickens and SLD chickens with normal chickens revealed that the *IGF2BP3* and *NDUFB2* genes were upregulated and the *NDUFA8*, *NDUFA9*, *NDUFB1*, *NDUFB5*, *NDUFB6*, *NDUFB8*, *NDUFB9*, *NDUFB10*, *NDUFS4*, and *SDHA* genes were downregulated. Moreover, some differences were also observed in the male RSS chickens. In particular, the *NDUFA8*, *NDUFA9*, *NDUFB1*, *NDUFB5*, *NDUFB6*, *NDUFB8*, *NDUFB9*, *NDUFB10*, *NDUFS4*, and *SDHA* genes were upregulated and the *NDUFB2* genes were downregulated in the livers of male RSS chickens, which is contrary to the consequences of female RSS chickens except for *IGF2BP3* gene (**Figure 5**). These results suggest that the *IGF2BP3* and *SDHA* genes, as major genes related to growth and energy supply, are associated with the development of RSS in chickens.

### Mitochondrial Function Measurement

**Figure 6:**
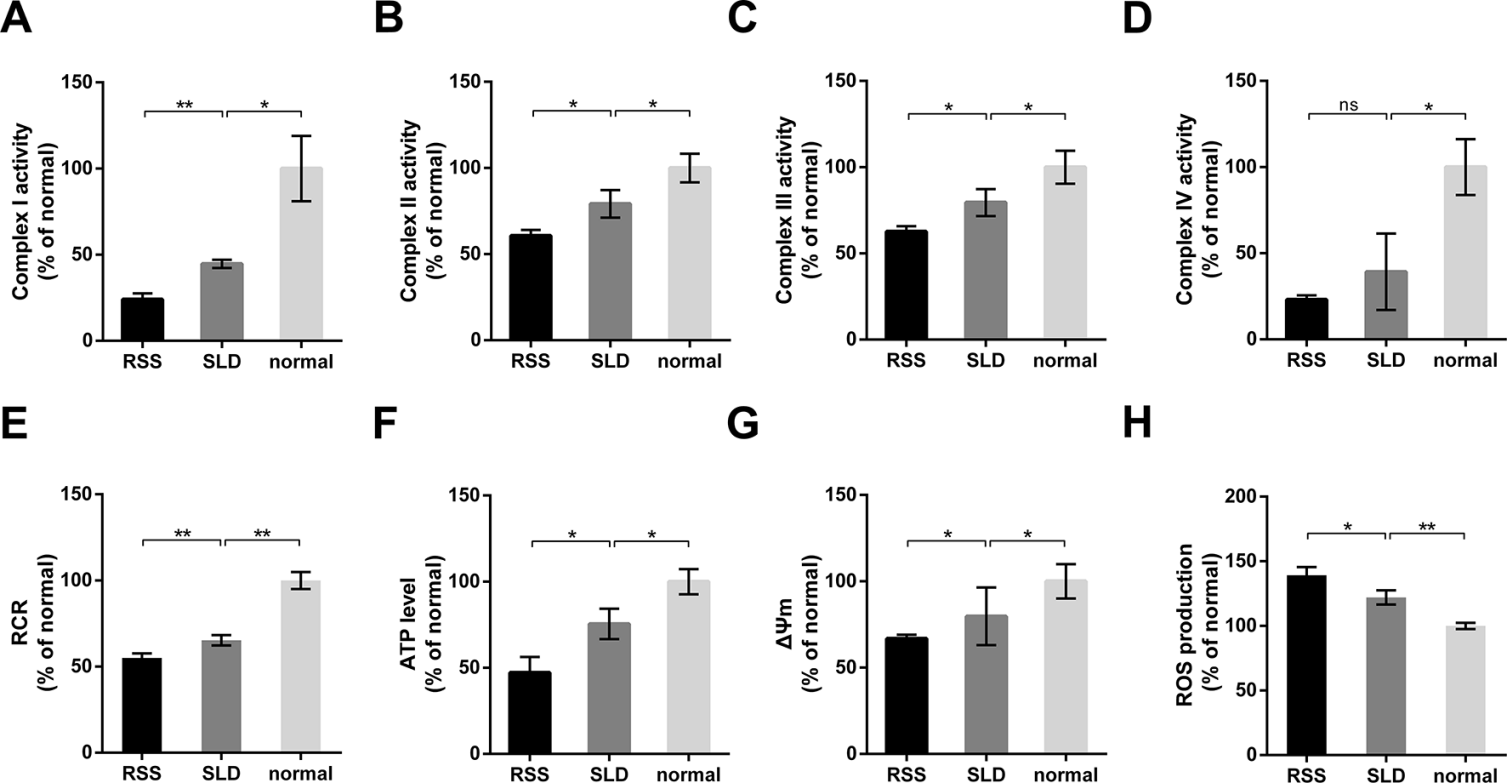
Enzymatic activities of mitochondrial respiratory complexes and mitochondrial function analysis of 7-week-old runting and stunting syndrome (RSS) (RSS) chickens, sex-linked dwarf (SLD) chickens, and normal chickens. The enzymatic activities of **(A)** complex I, **(B)** complex II, **(C)** complex III, and **(D)** complex IV in the livers of RSS chickens, SLD chickens, and normal chickens expressed a percentage of normal chickens. Respiratory control ratio (RCR) **(E)**, adenosine triphosphate (ATP) level **(F)**, ΔΨm **(G)**, and reactive oxygen species (ROS) production **(H)** in the livers of RSS, SLD, and normal chickens expressed as a percentage of normal chickens. Data are represented as the mean ± SEM; *p < 0.01; **p < 0.01; ns, no significant diffrerence.

The activities of mitochondrial enzymes in the liver, including OXPHOS complexes, are summarized in **Figure 6**. The enzyme activities of complex I, complex II, complex III, and complex IV in the RSS chickens were reduced by 45%, 24%, 21%, and 41%, respectively, compared with SLD chickens (**Figures 6A**–**D**). Similarly, the enzyme activities of complex I, complex II, complex III, and complex IV in the SLD chickens were reduced by 56%, 22%, 21%, and 61%, respectively, compared with normal chickens. To determine whether these changes in the activities of the respiratory complexes influenced the function of the mitochondria, we next assessed the mitochondrial function by measuring the RCR, ATP level, ΔΨm, and ROS production. The RCR values of the RSS chickens was reduced by 15% compared with SLD chickens, and that of SLD chickens was reduced by 35% compared with normal chickens (**Figure 6E**), indicating a lower oxygen consumption in the RSS chickens and SLD chickens. The ATP level of the RSS chickens was reduced by 37% compared with SLD chickens, and that of SLD chickens was reduced by 25% compared with normal chickens (**Figure 6F**), indicating a lower efficiency of ATP production in the RSS chickens and SLD chickens. The ΔΨm measured by JC-1 of the RSS chickens was reduced by 16% compared with SLD chickens, and that of SLD chickens was reduced by 21% compared with normal chickens (**Figure 6G**), indicating a dissipative ΔΨm in the RSS chickens and SLD chickens. Finally, the production of ROS in the RSS chickens was increased by 14% compared with SLD chickens, and that of SLD chickens was increased by 22% compared with normal chickens (**Figure 6H**).

## Discussion

The signs of RSS are usually observed in meat-type chickens between 3 and 6 weeks of age. Previous work has demonstrated that certain strains of birds appear to be more susceptible to the effects of RSS than others, and that male birds are more severely affected than females (Zavala, 2006). However, it is interesting to note that RSS-resistant broiler strains have stronger immunological responses against bacterial and viral infections than RSS-susceptible strains (Rebel et al., 2006). Some researchers have suggested that the poor growth and retarded feathering consistently observed in RSS-affected birds are due to a chronic infection. However, we found that SLD chickens were more susceptible to the effects of RSS and exhibited weaker immunological responses than normal chickens. Therefore, we studied 7-week-old yellow-feather broilers with RSS like features. Of note, No indication of viral was also detected in the liver tissues of the RSS chickens by viruses detection as previously described (Zavala, 2006; Zhang et al., 2015).

Further, we revealed that the mRNA of the insulin-like growth factor 2 mRNA-binding protein 3 (*IGF2BP3*) was upregulated in the RSS chicken's liver tissue. IGF2BP3 plays an important role in the formation and growth of many tumors and is typically expressed in embryonic tissue, muscles, and the placenta. In fact, the expression of *IGF2BP3* is low in the liver tissues of normal chickens. IGF2BP3 is an oncofetal protein with high expression during embryogenesis, low expression in adult tissues, and re-expression in malignant tissues (Palanichamy et al., 2016a). Another study revealed that IGF2BP3, as a human ontogenic master switch, restricts megakaryocyte development by modulating a lineage-specific P-TEFb activation mechanism, revealing potential strategies toward enhancing platelet production (Elagib et al., 2017). In this study, the expression of *IGF2BP3* in RSS chickens was examined by qRT-PCR. We found that *IGF2BP3* expression was increased in the livers of RSS chickens and SLD chickens. This raises the question of the nature of the association between enhanced *IGF2BP3* expression and RSS in chickens. Minchenko et al. demonstrated that the *IGF2BP3* gene is expressed in hypoxic tissues, indicating its participation in the regulation of metabolic and proliferative processes *via* IGF/INS receptors (Minchenko et al., 2015). In this study, we found that both the livers of RSS and SLD chickens exhibited signs of mitochondrial dysfunction, and the livers exhibited black or yellow in color. This suggests that the livers of RSS chickens are hypoxic. This would explain why *IGF2BP3* expression was increased in the livers of the RSS chickens. The RCR results further demonstrated that the livers of RSS chickens were hypoxic compared with SLD chickens. The abnormal expression of *IGF2BP3* confirmed that oxidative respiratory dysfunction occurs in the mitochondria of both RSS chickens and SLD chickens. Under hypoxic conditions, NADH and FADH_2_ cannot participate in respiratory chain regeneration, thereby interrupting the OXPHOS and preventing the normal synthesis of ATP. Our results also demonstrated that the ATP production was reduced in the livers of RSS chickens.

Moreover, we also revealed that the amounts of several components of the OXPHOS pathway in RSS chickens. *NDUFA8*, *NDUFA9*, *NDUFB1*, *NDUFB5*, *NDUFB6*, *NDUFB8*, *NDUFB9*, *NDUFB10,* and *NDUFS4* are downregulated in female RSS chickens compared to SLD chickens. However, the expression of these genes of female RSS chickens is contrary to that of male RSS chickens, which may indicate that the regulatory mechanisms of male and female RSS chickens are different. In particular, succinate dehydrogenase complex subunit A (SDHA) was abnormal expression in the livers of RSS chickens compared with those of SLD chickens. SDHA is anchored to the inner mitochondrial membrane by one hydrophobic subunit of SDHC (Sun et al., 2005). The catalytic subunit of SDHA is hydrophilic and extends into the mitochondrial matrix (Lancaster and Simon, 2002). SDHA contains a covalently attached flavin adenine dinucleotide (FAD) cofactor and the succinate-binding site. Flavination is important for succinate oxidation (Robinson et al., 1994). A previous study confirmed that flavination defects interfere with SDHA activity, likely reducing the overall growth rates *in vitro* and *in vivo* that have been observed in experimental models of SDHA deficiency (Guzy et al., 2008). Another study demonstrated that knockdown of the SDHA/B genes in HeLa cells led to the accumulation of succinate (Xiao et al., 2012). Konieczna et al. found that partial silencing of *SDHA* with specific siRNAs resulted in either less efficient or delayed entrance of the cells to the S phase (Konieczna et al., 2015). However, the cells remained viable. The results of this study revealed that *SDHA* was abnormal expression in the livers of RSS chickens compared with SLD chickens, but no mutation was detected. Lower OXPHOS activity will lead to insufficient energy supply, which appears to be one of the reasons for the development of RSS in chickens.

Decreased OXPHOS activities, along with higher ROS production in RSS and SLD chickens, are indicative of damaged mitochondrial function and efficiency. The vacuolar hydropic degeneration may induce the enlargement of hepatocytes and compress adjacent organelles, leading to damage of the nuclei and mitochondria of liver cells. This phenomenon was observed not only in the RSS chickens but also in SLD chickens with *GHR* mutation. Previous studies have confirmed that growth hormone (GH) stimulates cellular oxygen consumption in CHO cells transfected with cDNA coding for the full-length *GHR* (Perret-Vivancos et al., 2006a). GH affects mitochondrial metabolism by indirectly regulating the activities of OXPHOS enzyme complexes (Brown-Borg et al., 2012). We found that the abnormal interaction between GH and GHR can influence mitochondrial oxidation and respiratory functions. The five multimeric enzyme complexes that drive OXPHOS and cellular respiration are contained within the inner mitochondrial membrane (Barrientos et al., 2009). Determining the activities of individual enzymes in the respiratory chain complex provides an important diagnostic indicator of mitochondrial disease (Frazier and Thorburn, 2012). In mitochondrial disease, the activities of single or multiple complexes are reduced in the affected tissue (El-Hattab and Scaglia, 2013). Activity assays typically reveal decreased activities of complex I, complex III, and complex IV in liver tissues (El-Hattab and Scaglia, 2013). In this study, we observed decreased enzyme activities of complex I, complex II, complex III, and complex IV in RSS and SLD chickens.

Inefficient mitochondrial function was also observed upon measuring the RCR, ATP level, ΔΨm, and ROS production. As mitochondria are responsible for the majority of oxygen consumption, the RCR is an essential parameter for studying mitochondrial function (Frezza et al., 2007). Meanwhile, ΔΨm is essential for mitochondrial function, and mitochondrial dysfunction is normally accompanied by a decrease in ΔΨm (Javadov et al., 2006). Further, excessive ROS production can impair the activities of mitochondrial respiratory complexes and reduced ATP production (Sudheesh et al., 2013). Here, we observed alterations in the RCR, ATP level, ΔΨm, and ROS production in both RSS chickens and SLD chickens. However, the mitochondrial function was lowest in RSS chickens, indicating further mitochondrial dysfunction in RSS chickens compared with SLD chickens. This may further impair immunity, causing greater mortality in RSS chickens.

Mitochondria, the only organelles in animal cells that contain their own genome, exchange Ca^2+^ and ROS with the endoplasmic reticulum (Pacher and Hajnoczky, 2001; Zorov et al., 2014; Booth et al., 2016). An evolutionarily conserved machinery enables the complete and sequential fusion of the outer and inner mitochondrial membranes (Robinson et al., 1994). In this study, no inflammation was detected by H&E staining and no lymphocyte infiltration was observed. Vacuolation of hepatocytes was observed, and the cytoplasm was disappeared in RSS and SLD chickens. The hydropic degeneration in the hepatocytes was the main cause of the liver cell vacuoles. *GHR* mutation induces an abnormal interaction between GH and GHR. As *GHR* gene is associated with oxidative respiration (Perret-Vivancos et al., 2006b), this may further influences mitochondrial oxidation and respiratory functions. Here, the TEM results revealed that the inner and outer mitochondrial membranes of the RSS chickens exhibited varying degrees of damage or had even disappeared. Consequently, the five multimeric enzyme complexes within the inner mitochondrial membrane are unable to drive OXPHOS and cellular respiration. The *GHR* mutation, along with contributions from the additional factors mentioned above, may lead to the hindered growth and development in SLD chickens. If mitochondrial damage becomes more severe in SLD chickens, these may exhibit the signs of RSS. The methods reported in this study can also be used as a model for inner mitochondrial membrane *GHR* mutation.

Together, all of these changes in mitochondrial functions could account for mitochondria dysfunction, resulting in poor weight gain and retarded growth or stunting of chicks. Our findings revealed that the RSS in chicken is caused by mitochondria dysfunction. We didn't find mtDNA mutation in RSS and SLD chickens. The *GHR* mutation may cause mitochondria dysfunction in SLD chicken which is induced by *GHR* mutation, but RSS chicken's mitochondria dysfunction is more serious. We hypothesize that RSS chicken's mitochondrial disorders are caused by other nuclear gene mutations.

## Ethics Statement

All procedures involving animals were approved by South China Agriculture University's Institutional Animal Care and Use Committee (approval number SCAU#0017), according to the regulations established by this committee. Animals involved in this study were humanely sacrificed as necessary.

## Author Contributions

HL and BH contributed equally to this manuscript. HL designed the study, and wrote the paper. BH carried out experiments, and analyzed data. QL and SH participated in the design of the experiment and data analysis. YLu, BZ, and YG participated in data collection and interpretation, and helped with performing some of the manuscripts' experiments. YLi, MS, and QN helped for useful discussion and language correction. XZ and DZ participated in the design, manuscript writing and final approval of the manuscript. All authors read and approved the final manuscript.

## Funding

This work was supported by grants from the National Natural Science Fund Committee National Youth Project (Project grant no: 31401046), Guangdong Provincial Promotion Project on Preservation and Utilization of Local Breed of Livestock and Poultry and Guangdong Youth Talent Project.

## Conflict of Interest

The authors declare that the research was conducted in the absence of any commercial or financial relationships that could be construed as a potential conflict of interest.
