# Supplementary material for: Runting and Stunting Syndrome Is Associated With Mitochondrial Dysfunction in Sex-Linked Dwarf Chicken
Source: Front Genet. 2020 Jan 17;10:1337. doi: 10.3389/fgene.2019.01337 (PMC6978286; doi:10.3389/fgene.2019.01337)
Supplement: Supplementary file 1 [file Image_1.pdf]

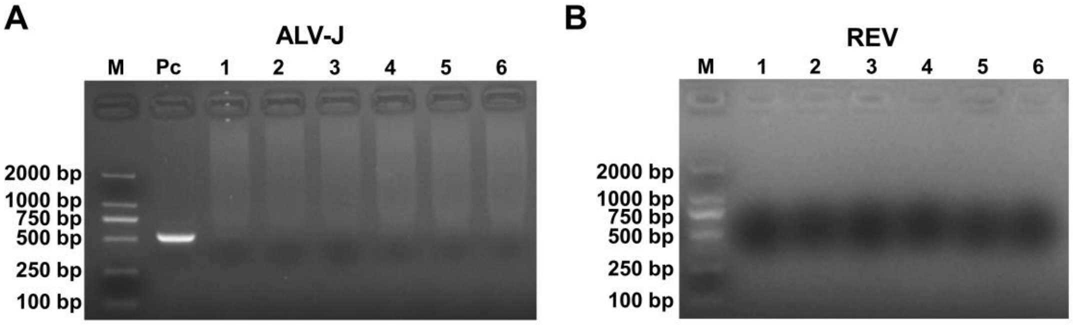


**Figure S1. The ALV-J and REV viruses detection of RSS chickens.** (A, B) All RSS chickens (1-6) were ALV-J and REV virus negative. M, DL 2000 marker. Pc, positive control.
